# Uniportal video-assisted thoracoscopic segmentectomy for fetal adenocarcinoma lung cancer with severe pulmonary emphysema: a case report

**DOI:** 10.3389/fonc.2024.1281211

**Published:** 2024-04-02

**Authors:** Yun-Sheng Wu, Yu-Ting Chen, Jen-Hao Chuang, Hsien-Chi Liao

**Affiliations:** ^1^ Department of Medical Education, National Taiwan University Hospital, Taipei, Taiwan; ^2^ Department of Pathology, National Taiwan University Cancer Center, Taipei, Taiwan; ^3^ Division of Thoracic Surgery, Department of Surgery, National Taiwan University Hospital, Taipei, Taiwan; ^4^ Department of Traumatology, National Taiwan University Hospital, Taipei, Taiwan; ^5^ Graduate Institute of Clinical Medicine, National Taiwan University College of Medicine, Taipei, Taiwan

**Keywords:** fetal adenocarcinoma, lung adenocarcinoma, uniportal video-assisted thoracoscopic, pulmonary emphysema, case report

## Abstract

**Background:**

Fetal adenocarcinoma is a very rare subtype of lung adenocarcinoma. Its incidence ranges from 0.1 to 0.87% among all primary lung neoplasms. Low-grade types tend to appear in the younger generation, and the age ranges from 20 to 50 years with a mean age of around 35 years. Surgical resection is currently the best way to treat fetal adenocarcinoma lung cancer without distant metastasis.

**Case report:**

This is a 56-year-old female who underwent low-dose computer tomography (LDCT) screening during the health examination. She used to be a heavy smoker for more than 30 years, and the CT images revealed severe bronchiectasis and emphysema. There is a solitary nodule with a diameter of 18.9 x 17.8mm in the central area of the left upper lobe. We decided to conduct left upper lobe S1~S3 segmentectomy under uniportal VATS. The surgery was successful, and the patient was discharged within one week and recovered well. The final diagnosis was fetal adenocarcinoma, low-grade (pT1cN0Mx, stage IA3).

**Conclusion:**

The first case reported as fetal adenocarcinoma lung cancer who underwent uniportal video-assisted thoracoscopic segmentectomy. We believe it is a safe and feasible procedure for low-grade types fetal adenocarcinoma patient with poor pulmonary function.

## 1 Introduction

Fetal adenocarcinoma is a very rare subtype of lung adenocarcinoma. Among all primary lung neoplasms, its incidence ranges from 0.1 (1) to 0.87% (2, 3). It is referred to as fetal adenocarcinoma because its tissue architecture and cell characteristics resemble fetal lung in 5−17 weeks of gestation (pseudoglandular stage). It was first considered the same disease as pulmonary blastoma (PB) in 1982 (4). However, since it lacks the mesenchymal components and has a completely different prognosis from PB, fetal adenocarcinoma was later on categorized as a variant of lung adenocarcinoma by the World Health Organization (5).

Microscopically, fetal adenocarcinoma consists of a complex glandular structure with glycogen-rich, non-ciliated cell linings. The cells have clear cytoplasm and characteristics of supranuclear or subnuclear vacuoles. Squamoid morules and fibroblastic stroma can be seen in the background (6). According to its histological patterns, it can be further divided into two groups: low-grade and high-grade types. Low-grade types show low nuclear atypia with frequent squamoid morules, which have pure patterns. In contrast, high-grade types exhibit prominent nuclear atypia, literally with few squamoid morules (3). Furthermore, other subtypes of lung adenocarcinoma usually present at the same time (7). Immunohistochemically, both low-grade and high-grade types show thyroid transcription factor 1 (TTF-1) positivity. On the other hand, beta-catenin is also related to fetal adenocarcinoma. In fact, studies demonstrated that morules and morule-like carcinomas in different organs were related to beta-catenin gene mutation (8). In low-grade types, tumor cells express abnormal nuclear and cytoplasmic staining of beta-catenin. As for high-grade types, these are not the cases. Another gene that can differentiate the two subtypes is *p53*, which is frequently mutated in high-grade types and usually absent in low-grade types (3).

Like other types of lung adenocarcinomas, fetal adenocarcinoma has those unspecific symptoms, such as cough, chest pain, pleural effusion, and so on. However, with the improvement of medical imaging tools, most cases are detected in the early stage and are diagnosed by histopathological findings. These clinical symptoms are, therefore, less important now; nevertheless, the clinical patterns of low-grade and high-grade types are different according to previous studies. Low-grade types tend to occur in young people aged 20−50 years, with a mean age of approximately 35 years (3, 9, 10). In contrast, high-grade types occur in older adult patients aged 50−75 years, with a mean age of approximately 65 years. Smoking history is highly related to high-grade type, with more than 90% of patients having smoking history (3, 7, 11–13). In low-grade types, lymphadenopathy, metastasis, and tumor recurrence are related to survival, but rarely occur. Surprisingly, tumor size is not related to prognosis. Moreover, the 5-year survival rate is >80% (17/21) (14). As for high-grade types, the prognoses are worse than the former because the disease usually presents symptoms in the later stage (3).

## 2 Case report

**Figure 1:**
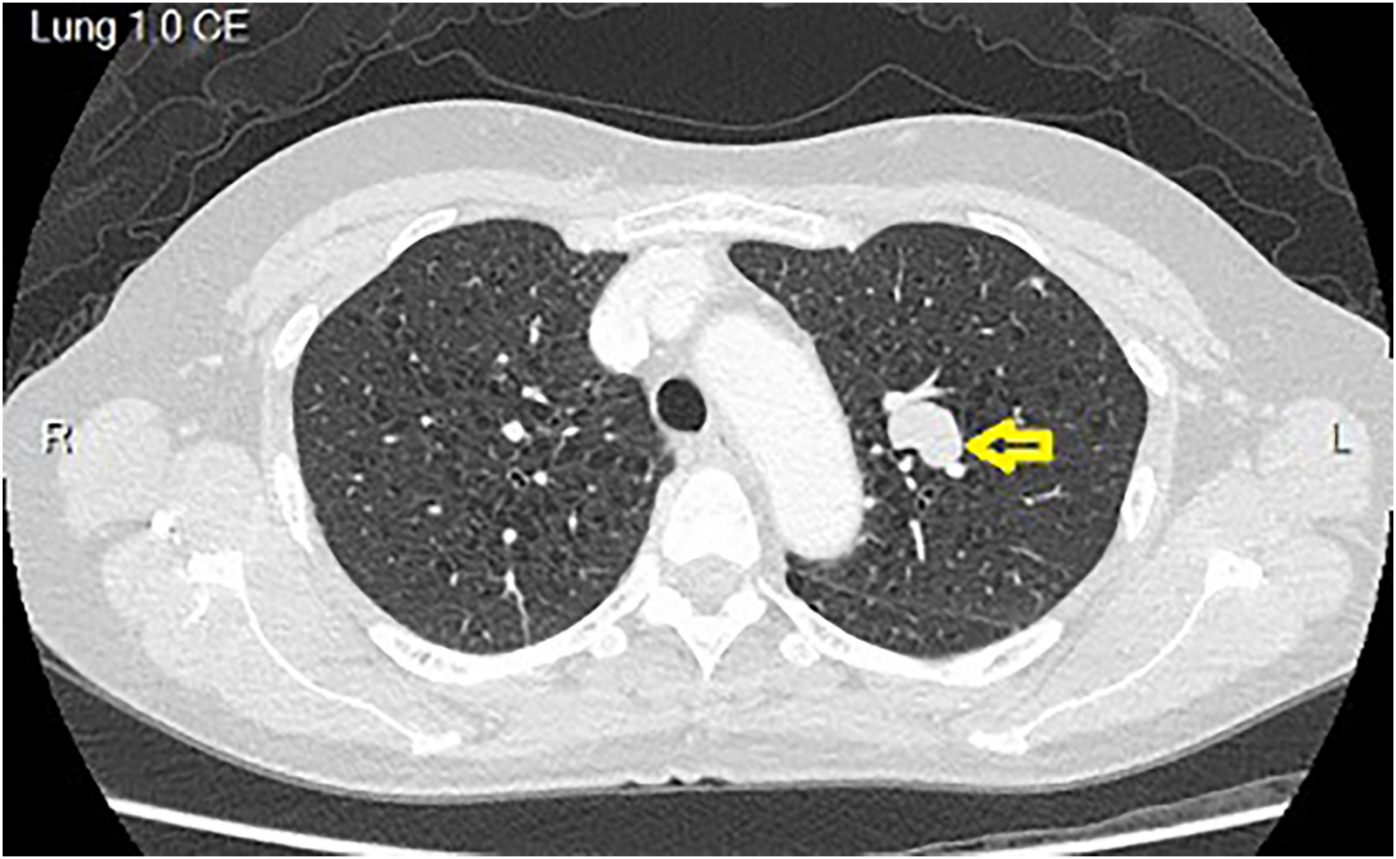
CT images revealed a solitary nodule with a diameter of 18.9 × 17.8 mm in the left upper lobe. The tumor is located in the hilum area. Besides, the CT images showed severe emphysematous change over bilateral lung parenchyma. CT, computed tomography.

A 56-year-old woman was found to have bilateral lung nodules on low-dose computer tomography (CT) during a routine medical examination and was referred to our hospital. She denied any discomfort, such as cough, sputum, chest pain, or body weight loss. The patient has no underlying disease and has a family history of liver cancer (father). Most importantly, she used to be a heavy smoker for 30 years. On chest radiograph, an abnormal shadow was noticed in the left upper lobe. CT images revealed a solitary nodule with a diameter of 18.9 × 17.8 mm in the left upper lobe (**Figure 1**). Besides, severe bronchiectasis was found on CT as well. Therefore, she was admitted to National Taiwan University Cancer Center and underwent pulmonary function test and cardiac sonography. The results showed forced expiratory volume in the first second of 2.41 L, which is 115.2% as predicted, and good left ventricle contractility with left ventricle ejection fraction of 69.6%.

**Figure 2:**
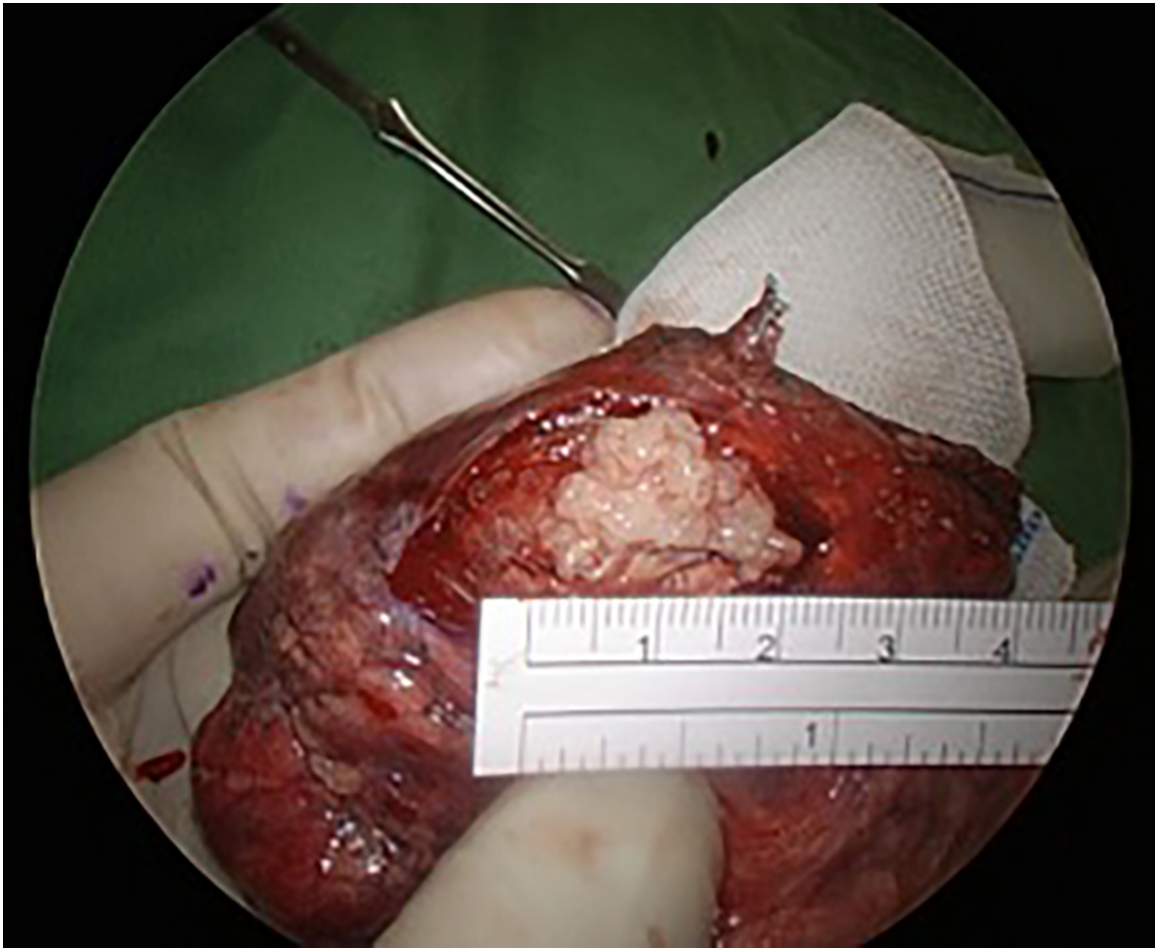
Grossly, the tumor was yellowish, well-defined, soft-to-elastic in texture tumor, and 21 × 15 × 14 mm in size.

**Figure 3:**
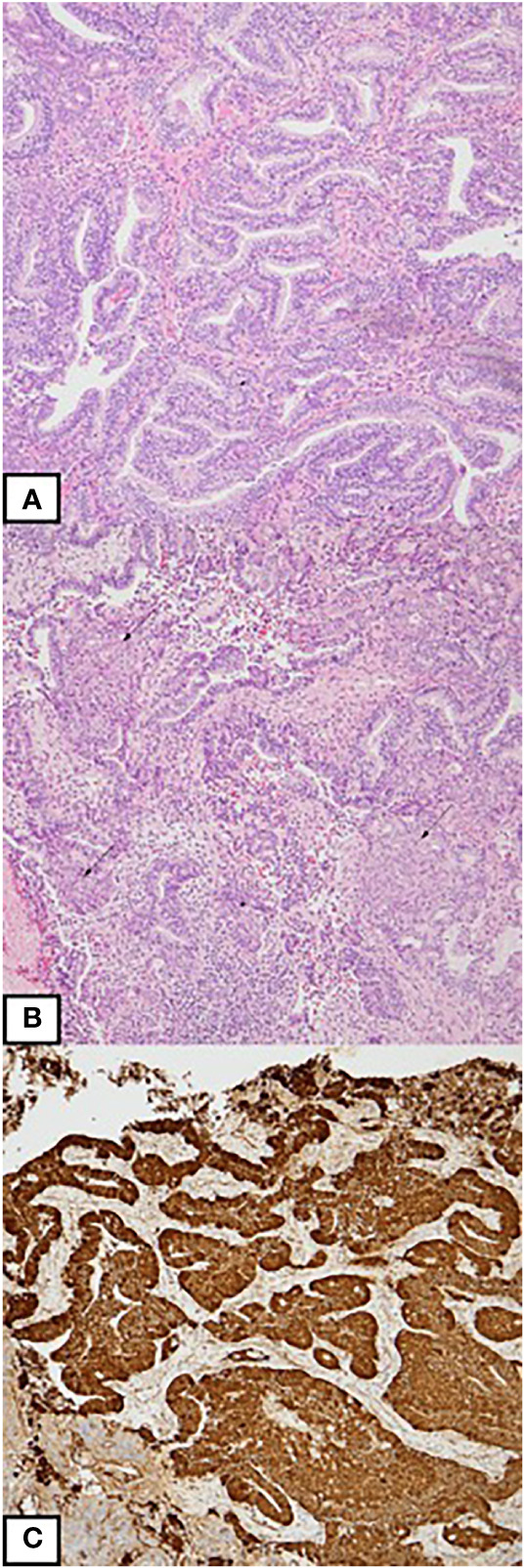
**(A)** An adenocarcinoma with complex glandular architecture. The tumor cells are columnar-shaped with vacuolated cytoplasm. **(B)** Morules formation is present (arrow). **(C)** Immunohistochemically, the tumor cells reveal aberrant nuclear expression of beta-catenin.

A left upper lobe S1-S3 segmentectomy and groups 5, 6, and 11 lymph node dissection were performed under uniportal video-assisted thoracoscopic (VATS) surgery. The intraoperative frozen biopsy showed adenocarcinoma with inflammatory cell-rich background. Grossly, the tumor was yellowish, well-defined, soft-to-elastic in texture tumor, and 21 × 15 × 14 mm in size (**Figure 2**). The pathological findings showed a complex glandular structure with frequent squamoid morules. The tumor cells have basally oriented nuclei and vacuolated cytoplasm. In addition, small foci of fibroblastic stroma are identified focally (**Figure 3**). Immunohistochemical stains showed TTF-1 positivity, CK (AE1/AE3) positivity, CDX-2 negativity, PAX8 negativity, and nuclear stain on beta-catenin. The low-grade fetal adenocarcinoma of lung origin was favored. Next-generation sequencing revealed no G719X and Exon 19 deletions, S768I, T790M, and Exon 20 insertions, and L858R and L861Q EGFR mutation.

No lymph node metastasis was detected. Moreover, both the postoperative follow-up of brain magnetic resonance imaging with/without contrast and whole-body fluorodeoxyglucose-positron emission tomography showed no distant metastasis. The final diagnosis was low-grade fetal adenocarcinoma (pT1cN0Mx, stage IA3).

## 3 Discussion

This is the first case of a patient with fetal adenocarcinoma lung cancer who underwent uniportal video-assisted thoracoscopic segmentectomy. In our hospital, we perform over 1500 lung tumor surgeries annually. Even with such a high volume, cases of fetal adenocarcinoma are still rare. In this case, the patient had no symptoms, and the lesion was found was found during routine imaging investigation.

According to studies, lobectomy remains the standard of care for tumors 2−3 cm in size (15, 16). However, because the tumor was closer to the upper tri-segment area of the lung, and it was harder it is to remove the tumor under wedge resection. Both characteristics of tumor size and location point to lobectomy. A tri-segment approach was adopted because patient could not afford lobectomy, because there is no evidence in the data provided of severe emphysema or other severe comorbidities that would prohibit major lung resection. According to Chan et al., the recurrence-free or overall survival at 5 years between segmentectomy and lobectomy for patients without nodal disease (AJCC 8th Edition Stage 1A NSCLC) showed no significant differences (17). Therefore, we decided to perform segmentectomy under uniportal VATS. Our surgical team believes uniport approach can provide enhanced outcomes (18). Some advantages meet our needs according to the patient status.

The prognosis of low-grade types is very good, especially those stage I cases, which can even be up to 90%. According to Sato et al., 22 cases were stage I disease among all resected 25 low-grade types cases. Among these stage I cases, three patients showed recurrence, and one died. However, all three patients with recurrence had tumor size >3 cm. As for tumors <3 cm, no recurrence or death was reported (1). Surgery is the standard treatment for low-grade type fetal adenocarcinoma. Some studies reported that chemotherapy did not result in long-term survival, but still prolonged survival (14). Another study demonstrated partial response of neoadjuvant chemotherapy in low-grade type fetal adenocarcinoma. However, the effects of chemotherapy still need further evaluation. In summary, surgical treatment and regular follow-up are safe and feasible for such patients (pT1cN0Mx, stage IA3).

## Data availability statement

The original contributions presented in the study are included in the article/supplementary material. Further inquiries can be directed to the corresponding author.

## Ethics statement

The studies involving humans were approved by National Taiwan University Hospital. The studies were conducted in accordance with the local legislation and institutional requirements. The participants provided their written informed consent to participate in this study. Written informed consent was obtained from the individual(s) for the publication of any potentially identifiable images or data included in this article. Written informed consent was obtained from the participant/patient(s) for the publication of this case report.

## Author contributions

Y-SW: Data curation, Writing – original draft, Writing – review & editing. Y-TC: Data curation, Writing – review & editing. J-HC: Data curation, Writing – review & editing. H-CL: Conceptualization, Data curation, Formal analysis, Writing – review & editing.
